# Imported Malaria in Portugal 2000–2009: A Role for Hospital Statistics for Better Estimates and Surveillance

**DOI:** 10.1155/2014/373029

**Published:** 2014-12-07

**Authors:** Ana Glória Fonseca, Sara S. Dias, João Luis Baptista, Jorge Torgal

**Affiliations:** ^1^Public Health Department, Nova Medical School, Nova Lisbon University, Campo Mártires da Pátria 130, 1169-056 Lisbon, Portugal; ^2^UIS-ESSLei-IPLeiria, Campus 2, Morro do Lena, Apartado 4137, 2411-901 Leiria, Portugal; ^3^Faculty of Health Sciences, Beira Interior University, Avenue Infante D. Henrique, 6200-506 Covilhã, Portugal

## Abstract

*Background*. Although eradicated in Portugal, malaria keeps taking its toll on travelers and migrants from endemic countries. Disease notification is mandatory but is compromised by underreporting. *Methods*. A retrospective study on malaria hospitalizations for 10 consecutive years (2000–2009) was conducted. Data on hospitalizations and notifications were obtained from Central Administration of Health System and Health Protection Agency, respectively. For data selection ICD-9 CM and ICD-10 were used: codes 084^*^, 647.4, and B50–B54. Variables were gender, age, agent and origin of infection, length of stay (LOS), lethality, and comorbidities. Analysis included description, hypothesis testing, and regression. *Results*. There were 2003 malaria hospitalizations and 480 notified hospitalized cases, mainly in young male adults. *P. falciparum* was the main agent of infection acquired mainly in sub-Saharan Africa. Lethality was 1.95% and mean LOS was 8.09 days. Older age entailed longer LOS and increased lethality. *Discussion*. From 2000 to 2009, there were 2003 malaria hospitalizations with decreasing annual incidence, these numbers being remarkably higher than those notified. The national database of diagnosis related groups, reflecting hospitalizations on NHS hospitals, may be an unexplored complementary source for better estimates on imported malaria.

## 1. Introduction

Outside endemic regions, malaria may occur in travellers that have recently been to or visited endemic regions or in individuals from endemic countries recently arrived at or visiting nonendemic regions. Imported malaria is therefore defined as “an infection that was acquired in an endemic area by an individual (either a tourist or indigenous native) but diagnosed in a nonendemic country after development of the clinical disease” [1].

The increase in international travel and climate changes are considered important causes for the emergence of imported malaria. International travel to malarial zones is estimated at 80–90 million travellers annually and results in an estimated 30000 annual imported malaria cases worldwide [2, 3].

Since the year 2000 and contrary to the trend in the preceding years, there has been a progressive and steady decrease in the annual incidence of imported malaria that varied from 15303 reported cases in the World Health Organization (WHO) European Region in 2000 to 5712 in 2009 [4]. Those promptly diagnosed and treated generally have a full recovery, making imported malaria an avoidable cause of death that nevertheless still accounts for a considerable preventable burden of morbidity and mortality every year in nonendemic countries.

WHO declared autochthonous malaria eradicated in Portugal in 1973 [5]. However, due to increasing international travel and travel related to extensive international cultural and commercial relations, malaria keeps taking its toll on travelers and migrants to and from endemic countries. Disease surveillance consists of a physician based passive case detection system liable to underreporting. National data on imported malaria are scarce or nonexistent, the available data being based on sporadic single hospital based or regional based case series and case reports [5–9].

Complementing notification data may allow for better malaria burden estimates. Imported hospitalized malaria cases from 2000 to 2009 were analysed at national level, using Portugal's Diagnosis Related Groups (DRG) information system from National Health Services (NHS) hospital episodes statistics and Statutory Notification information system. Frequency of occurrence, hospital length of stay, lethality, plasmodium species, and origin of infection were considered. Secondly, the influence of demographic factors and comorbidities on in-hospital length of stay and lethality were analyzed.

## 2. Methods

A retrospective study was conducted, using Portuguese National Diagnosis Related Groups (DRG) database, resulting from the NHS hospital episodes statistics, and the National Statutory Notifiable Disease Surveillance database, resulting from disease notification, provided by the Central Administration of Health System (ACSS) and the Health Protection Agency (HPA), respectively. The data were made anonymous for analysis.

In the DRG database each record corresponds to a National Health Service (NHS) hospital discharge episode (hospitalization). The Diagnostic Related Groups (DRG) were developed as an inpatient classification system that determines the payment allocated to the hospital [10]. It provides a complete record of all hospitalizations and is not compromised by the limitations of existent surveillance systems, such as under diagnosis or deficiencies in reporting. Within DRGs the 9th International Classification Disease Clinical Modification (ICD-9 CM) is used to classify all diagnosis assigned by the physician at discharge. Malaria diagnosis implies laboratorial microscopic parasite detection and/or antigen parasite detection test for* Plasmodium falciparum* and nonfalciparum infections. Molecular PCR technology is not widely available but is possible in some institutions, mainly those involved in research.

Malaria surveillance, on the other hand, is a passive mandatory physician based case detection system in Portugal resulting in the National Statutory Notifiable Disease Surveillance database [11]. A standardised paper notification form, equal to all notifiable diseases, is used. During the period of study only confirmed cases were notified. There is no laboratory based notification system. In the National Statutory Notifiable Disease Surveillance database each record corresponds to a case of disease. The 10th International Classification Disease (ICD-10) is used to classify diagnosis.

The study population was selected using ICD-9 CM, codes 084 (malaria) [namely 084.0 (*Plasmodium falciparum*), 084.1 (*Plasmodium vivax*), 084.2 (*Plasmodium malariae*), 084.3 (*Plasmodium ovale*), 084.5 (mixed malaria), 084.6 (malaria unspecified), and 084.9 (pernicious complication)] and 647.4 (malaria in the mother classifiable elsewhere, but complicating pregnancy, childbirth, or the puerperium), for the DRG database and 10th International Classification Disease (ICD-10), codes B50 (*Plasmodium falciparum*), B51 (*Plasmodium vivax*), B52 (*Plasmodium malariae*), B53.0 (*Plasmodium ovale*), and B54 (malaria unspecified), for the National Statutory Notifiable Disease Surveillance database.

Data on malaria cases is nationwide and covers a 10-consecutive-year study period (2000–2009). Variables from de DRG database included gender, age, year of hospitalization, month of hospitalization, district of patient's residence, in-hospital length of stay (LOS), and lethality. Variables from the statutory notification database included year of notification, agent and origin of infection, and lethality. Certain characteristics, such as nationality and whether they are travelers, immigrants, expatriates, or visiting friends and relatives (VFRs), treatment schedule, chemoprophylaxis use, and duration of travel in endemic countries, could not be retrieved from the databases because these items are not systematically collected.

In the DRG database, potential factors influencing LOS or death were defined: (a) age > 64 years; (b) gender; (c) malaria related to pregnancy (ICD-9 code 0647.4); (d) HIV infection (ICD-9 code 042); (e) other infections excluding malaria (ICD-9 codes 001–009, 011–018, 020–027, 030–040, 042, 060–066, 070, 071, 082, 083, 085, 086, 091–095, 098, 100, 120–129, and 130); (f) pneumonia (ICD-9 codes 480–486); (g) respiratory failure (ICD-9 codes 518.81–518.84); (h) chronic obstructive pulmonary disease (ICD-9 codes 490–496); (i) diabetes mellitus (ICD-9 code 250); (j) arterial hypertension (ICD-9 codes 401–405); (k) renal failure (ICD-9 codes 584, 585, 586, 403, and 404); (l) anemia (ICD-9 codes 280–285); (m) thrombocytopenia (ICD-9 codes 287.4-287.5).

The demographic characteristics of the patients were summarised using mean and standard deviation (SD) and medians and interquartile range (IQR) for continuous data and using proportions for categorical data. Hypothesis testing was used to compare groups: *t*-tests and ANOVA, assuming normal distribution for continuous data; chi-square test or Fisher exact test (if the expected values were too small) for categorical data. Linear regression model was used to study the length of stay and logistic regression model used to study the effect on lethality. Due to the skewed length of stay data, a log transformation was carried out to normalise the data before multivariate analysis; this procedure is usual for such data [12, 13]. Although gender and age are not statistically significant at the 5% level, they were retained in the model in order to control for possible confounding. Covariates controlled in multivariate analysis were the potential factors defined before. The effect sizes from the multivariate analysis are reported as relative ratios (exponential of the regression coefficient of the log transformed data) for the in-hospital length of stay and as odds ratio (OR) for lethality. Confidence intervals are given at 95% and *P* values less than 0.05 are statistically significant, although the multivariate final models include covariates with 10% significance level in the univariate analysis. Data were analysed using R software, namely, glm library.

## 3. Results

**Figure 1:**
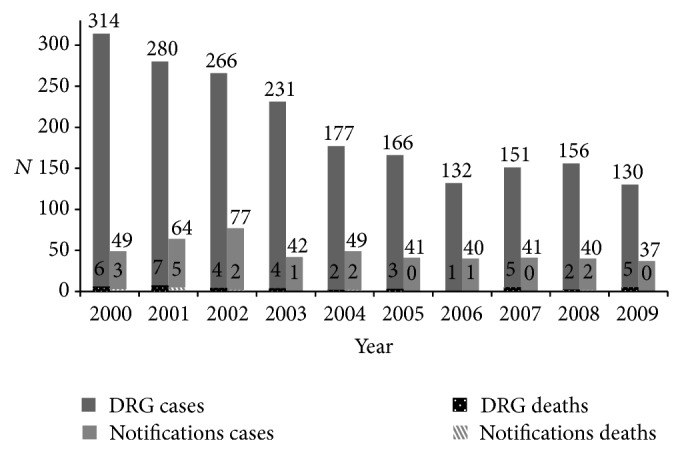
Imported malaria hospitalizations and deaths (NHS DRG database) and notifications of hospitalized cases and deaths (Statutory Notifiable Disease database) in Portugal 2000–2009.

Table 1 summarizes the general descriptive demographic and epidemiological characteristics, considering hospitalizations and notifications of hospitalized cases.  Figure 1 shows the overall annual trends in malaria cases and deaths. The two databases were analysed separately.

### 3.1. Imported Malaria Hospitalizations at NHS Hospitals (DRG Database)

From 2000 to 2009, there were 2003 hospitalizations with malaria diagnosis, the annual incidence declining from 314 hospitalizations in 2000 to 130 in 2009. Most cases were male (1416, 71%) aged 18–64 years (1650, 82.4%, mean age 37.7 years), with residence at Lisbon (986, 49.2%), Setubal (169, 8.4%), and Porto (259, 12.9%). In-hospital mean length of stay (LOS) was 8.09 days and lethality was 1.95% (39/2003).

In the first four years the annual number of malaria hospitalizations was significantly higher than in the following years (chi-square test *P* < 0.001). Mean length of stay remained stable throughout the years, varying from 6.88 days to 9.39 days, the annual variation not being significantly different (ANOVA test *P* > 0.05). Lethality remained relatively stable, ranging from 0.75% to 3.85%, with peaks in 2001 (2.5%), 2007 (3.3%), and 2009 (3.5%). Those who died had a significantly higher mean length of stay (20.5 days for those who died and 7.84 days for those who did not die, *t*-test, *P* < 0.001).

In January, May, June, and December (202, 193, 207, and 203 cases resp.), observed cases exceeded by 26 to 40 those expected. On the contrary, in February and March (132 and 126) the frequency of cases was 35 and 40 lower than expected (chi-square test *P* < 0.001). Mean length of stay ranged from 6.77 days in September to 9.32 days in April but no significant differences were observed (ANOVA *P* > 0.05). Absolute mortality and lethality were the highest in March (*n* = 5, 3.97%) and the lowest in September (*n* = 1, 0.64%).

**Figure 2:**
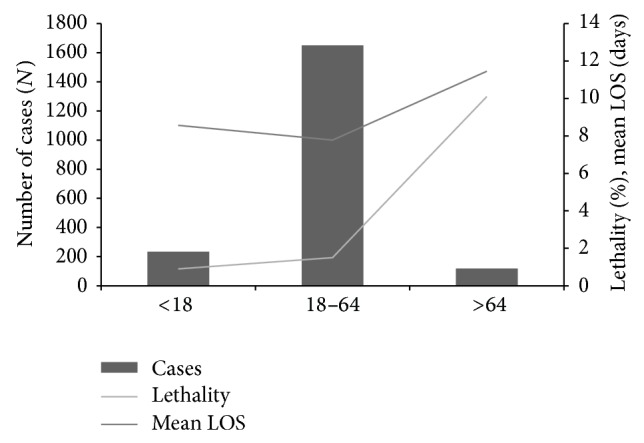
Overall effect of age (age category in years) on case frequency, mean length of stay (LOS), and lethality (DRG data 2000–2009).

On age analysis, more than 75–80% of annual hospitalizations were on those aged 18–64 years. Older age (>64 years old) corresponded to 5.9% (119) of all hospitalizations and younger age (<18 years old) corresponded to 11.7% (234). Older age (>64 years old) had significantly higher mean length of stay (11.45 days versus 7.88 days,* t*-test, *P* = 0.001) and lethality (10% versus 1.43%, Fisher's exact test, *P* < 0.001). Overall, malaria lethality increased steadily with increasing age (0.9% <18, 1.5% 10–64, 10% >64) (Figure 2). On gender analysis, the majority of annual hospitalizations were in males and no significant differences were obtained for mean length of stay (8.07 for male versus 8.15 for female, *t*-test, *P* > 0.05) nor for lethality (2.0% for male versus 1.7% for female, chi-square test, *P* > 0.05).

### 3.2. Imported Malaria Notifications of Hospitalized Cases (Statutory Notification)

Between 2000 and 2009, there were 541 confirmed malaria statutory notifications (480 hospitalized cases, 42 ambulatory cases, and 19 not known). From the 480 notifications referring to hospitalized cases, most were in males (376, 78.3%), mean age was 38.5 years, and in 16 cases outcome was death (3.33%). Within these (*n* = 480),* P. falciparum* was the most frequent agent of infection (*n* = 328, 68.3%) followed by* P. vivax* (*n* = 40, 8.3%). In 103 cases (21.5%) there was no species identification. Eight coinfections were identified: 5 with* P. falciparum* and* P. vivax*, 1 with* P. falciparum* and* P. ovale*, and 2 with* P. vivax* and* P. ovale*. Nine out of the 16 malaria deaths were due to* P. falciparum* and for the remaining deaths the species was not specified. In 73.3% of cases (*n* = 352) the identified origin of infection was sub-Saharan Africa.

### 3.3. Factors Influencing Length of Stay and Mortality (Tables 2 and 3)

Considering NHS malaria hospitalizations (*n* = 2003), the frequency of predefined comorbidities (age > 64, pregnancy related malaria, HIV infection, other infections excluding malaria, pneumonia, respiratory failure, chronic obstructive lung disease, diabetes, arterial hypertension, renal failure, anemia, and thrombocytopenia) ranged from 1.84% (malaria related to pregnancy) to 21.27% (anemia).

#### 3.3.1. Length of Stay (LOS)

The overall in-hospital mean length of stay was 8.09 days and the median was 6 (IQR 4–8). On univariate analysis, the following variables were associated with prolonged length of stay: age > 64 years, HIV infection, other infections, chronic obstructive lung disease, pneumonia, respiratory failure, renal failure, and anemia. Gender, diabetes, and arterial hypertension did not significantly increase mean of length of stay. This association prevailed on multivariate analysis for age, other infections, pneumonia, respiratory failure, and anemia. Compared to those without infection the adjusted relative ratio (RR) for other infections was 1.85 (95% CI 1.57–2.18) followed by pneumonia (RR = 1.82, 95% CI 1.51–2.21). Respiratory failure has a high RR in unadjusted and adjusted analysis, although it reduces after adjusting for possible confounding factors. On the contrary, age > 64 further increased length of stay when other factors were considered together.

#### 3.3.2. Lethality

The overall in-hospital lethality was 1.95% (39/2003). On univariate analysis, the following variables were associated with increased lethality: age > 64 years, HIV infection, other infections, diabetes, pneumonia, respiratory failure, and renal failure. This association prevailed on multivariate regression analysis for age > 64, respiratory failure, and renal failure. In comparison to those without respiratory failure the adjusted odds ratio was 32.79 (95% CI 13.16–81.70), followed by age > 64 years (OR = 5.37, 95% CI 2.14–13.50). The OR of the significant comorbidities decreased when the other factors were considered together.

## 4. Discussion

Between 2000 and 2009, there were 2003 malaria admissions to NHS hospitals in Portugal. The data show a consistent declining annual trend contrasting with a relative stability in mean length of stay and lethality observed in the same period. Overall in-hospital lethality was 1.95% and mean length of stay was 8.09 days.

This decreasing incidence of imported malaria despite increasing global international travel (malaria endemic regions included) is in contrast with the steady increase until the year 2000 and has been observed in other countries in Europe [4, 14, 15]. Reductions on malaria transmission have been achieved in many malaria endemic countries, as malaria control programmes have been successfully implemented [16, 17]. Nevertheless, malaria hospitalizations are known to have increased since 2009 in Portugal, increased expatriation to malaria endemic countries being one plausible explanation [18].

For the same time period (2000–2009), National Statutory malaria notification consisting mainly of hospitalized cases (480, 88.7% of total malaria notifications) only captured a quarter of total hospitalizations and indicated stable annual case frequency (40–50 per year), contradicting the declining trend observed for NHS hospitalizations. This denotes very high malaria underreporting, moreover considering that many malaria cases are treated without hospital stay and therefore are not included in the DRG database.

Malaria underreporting is not new but is worrying. Underreporting estimates of imported malaria in Europe ranged from 20 to 59% [19]. In Portugal there are no quantified estimates. Malaria is included in the National Notifiable Infectious Diseases Surveillance Programme, a passive clinician based mandatory surveillance system. The notification process is not very user friendly and the time involved in completing the different steps in the system compromises timeliness. It consists of manually filling in a specific notification paper form, common for all notifiable infectious diseases, and parasite confirmation is microscopic and/or by antigen detection test [1, 11]. There is no laboratory based notification, contrary to other European countries which have better reporting performances or improved them on including such notification [1, 20–23]. The notification paper forms are frequently not readily available in the institutions and have to be sent by post. As such, with the daily hustle bustle demands of patient care, clinicians may easily forget to do it, furthermore considering they might not be fully aware that malaria is included in the notifiable disease list or of the legal requirement to report. The use of a notification paper form, common to all notifiable infectious diseases, results in missing or omission of relevant epidemiological data specific to malaria. This is further aggravated by the fact that subsequent epidemiologic surveys of malaria cases and contacts are not routinely done. Therefore, relevant information such as time delay to diagnosis and treatment, duration and purpose of travel, drug treatment used, and personal protection measures is very difficult to gather. Contributing factors to underreporting have not been analysed in Portugal but excess work and lack of time, lack of familiarity with the list of notifiable diseases, lack of understanding of the importance of notification, and concerns regarding confidentiality have been identified elsewhere and can easily apply to Portugal [24, 25]. This study confirms that malaria underreporting is an issue and it needs to be thoroughly addressed to uncover its reasons and implement strategies to overcome it. Reporting timeliness delays, data quality and completeness of data, cumbersome reporting process, and lack of laboratorial notification, as discussed above, are other limitations to consider in addition to the scarcity of systematic malaria surveillance activities evaluation. Electronic Internet based reporting systems are considered more efficient with proven benefits in terms of timeliness and completeness compared to conventional systems and are already in use in some countries [26–28]. In Portugal, the implementation of the National Epidemiology Surveillance System, an electronic national surveillance system including infectious diseases and other public health risks, is starting and improvements in reporting performance are expected [29].

Cases showed a bimodal pattern with peaks in May-June and December-January not combined with mortality peaks. Sub-Saharan Africa was the main origin of imported infection in Portugal as observed in this and others studies [5, 6, 8].

Most cases occurred in working age male adults, a gender and age predominance observed in other European and non-European countries [3, 15, 30–32]. No male predominance was observed for length of stay or lethality though. Increasing age was strongly and independently associated with higher length of stay [adjusted-RR for age 1.29 (CI 1.12–1.50, *P* = 0.001)] and lethality [adjusted OR for age > 64 5.37 (CI 2.14–13.50, *P* < 0.001)]. These data are supported by previous reports of increased lethality of imported malaria in older age groups [33–35].

Statutory notification identified falciparum malaria as the most frequent agent of infection which is in agreement with sub-Saharan Africa as the most common geographical origin of infection. In 103 and 98 notifications, respectively, the agent and origin of infection were not stated, reflecting either reporting inaccuracies due to incomplete information or inability for specific or complete species identification in some hospitals, the latter due to the type of parasite detection method used.

Inpatient malaria diagnosis and treatment policy in Portugal follows WHO guidelines for malaria treatment, but drug treatment consists firstly of quinine plus doxycycline or quinine plus clindamycin [36]. IV artesunate or artemisinin-based combination therapies (ACT) are not widely available in hospitals but possible on request. Primaquine is used on vivax infections. ACT combinations, namely, dihydroartemisinin-piperaquine, are available for outpatient treatment. Mefloquine and atovaquone/proguanil are usually reserved for chemoprophylaxis [37].

Respiratory failure, renal failure, and severe anemia are among the WHO criteria for severe malaria, predicting higher risk of severe disease and death [38]. On comorbidity analysis respiratory failure was associated with higher length of stay and lethality, and renal failure was associated with higher lethality but not higher length of stay. Anemia increased length of stay but did not increase lethality. The data however do not allow a severity stratified analysis as other WHO criteria for severity could not be retrieved from DRG database. Pneumonia and other infections significantly increased length of stay but not lethality. Coinfections, namely, pneumonia and bacteraemia, community acquired or nosocomial, are not infrequent in hospitalized malaria, especially in severe cases, and may affect management and outcome [8, 39].

None of the chronic diseases analysed (HIV infection, chronic pulmonary disease, diabetes mellitus, and arterial hypertension) seemed to have an effect neither on length of stay nor on lethality, their prevalence ranging from 1.75% to 6.94%. The data on chronic diseases, such as diabetes mellitus and arterial hypertension, and on the severity of imported malaria are scarce. Their frequency does increase with increasing age though, and age is an independent risk factor for mortality as shown here and elsewhere [33–35]. Generally, the association of HIV infection and imported malaria has not consistently been related to worse outcome, its impact on malaria severity depending on the severity of the immunosuppression [40–43].

Malaria outcome depends on many factors, as retrieved from sentinel surveillance data, hospital based case series (hospital clinical records), and surveillance data analysis in countries with organized malaria surveillance programs. These include nationality, traveler characteristics (e.g., expatriate, immigrant, VFR, etc.), geographic origin, travel destination, adhesion to chemoprophylaxis, delays in seeking medical care, delays in diagnosis and treatment of suspected malaria, and treatment schedule that could not be included in multifactorial analysis because they are not included in the database [33, 44]. The results obtained however suggest that some comorbidities may prolong the length of stay and increase lethality.

There are limitations associated with the use of DRG data. The information on hospital characteristics was not gathered for epidemiological purposes; some vital features of malaria patients are missing and therefore it does not allow for the analysis of variables usually seen in epidemiologic studies. The DRG data cannot account for multiple admissions, reducing our experimental unit to discharge episode instead of patient. Its use however allows for obtaining timely nationwide data without the constraints and limitations of surveillance systems, such as under diagnosis or underreporting, providing data that would be otherwise unfeasible or logistically, cumbersome, difficult, and expensive to obtain.

Malaria is not easily subject to miscoding, due to diagnosis specificities (parasite detection by microscopy, antigen detection tests, or molecular PCR technology), though malaria subtypes may not be accurately or specifically discriminated on codification; therefore no such subtype analysis was done within the DRG database.

The use of hospital episode statistics, such as DGR database, for research and epidemiological purposes in infectious and noninfectious diseases, being able to congregate data at local and national level, is not original though results have to be analyzed considering the specificities of the disease under study, data quality and coding accuracy, and advantages and limitations of the database [45].

## 5. Conclusions

This is to our knowledge the first comprehensive national level study on imported malaria in Portugal and one that provides new insight on malaria burden estimates, particularly concerning malaria treated in hospitals, using the national databases available. Hospitalized malaria notifications reflected a quarter of NHS malaria hospitalizations and less than half of in-hospital lethality. Older age significantly increased hospital stay and lethality, and comorbidities are suggested as significant influencing factors.* P. falciparum* was the main plasmodium species responsible for the malaria cases and sub-Saharan Africa is the main geographic region of infection.

Along with climate change, international travel is thought to increase the risk of imported malaria, making malaria surveillance an important pillar of public health protection. Hospital statistics episodes (DRG database) analysis may be an unexplored complementary resource, in addition to statutory notification, for imported malaria surveillance in Portugal, especially considering the nonquantified underreporting, the increasing international travel, and the recent boost in emigration to malaria endemic regions in Portugal.

## Figures and Tables

**Table 1:** Demographic/general characteristics.

	NHS hospitalizations (DRG database)	Statutory hospitalized notifications
Gender (*n*, %)		
Female	587, 29%	376, 78.3%
Male	1416, 71%	104, 21.7%
Age (years)		
Mean	37.7	38.5
Median	38	38
Standard deviation	17.019	15.46
Minimum; maximum	0; 98	0; 85
Month of hospitalization (*n*, %)		—
January	202, 10.1%	
February	132, 6.6%	
March	126, 6.3%	
April	165, 8.2%	
May	193, 9.6%	
June	207, 10.3%	
July	161, 8.0%	
August	154, 7.7%	
September	156, 7.8%	
October	150, 7.5%	
November	154, 7.7%	
December	203, 10.1%	
Agent of infection (*n*, %)	—	
*Plasmodium falciparum *		328, 68.3%
*Plasmodium vivax *		40, 8.3%
*Plasmodium ovale *		5, 1.04%
*Plasmodium malariae *		5, 1.04%
*Plasmodium *sp. not specified		103, 21.4%
Origin of infection	—	
Sub-Saharan Africa		352, 73.33%
Central Continental America		1, 0.21%
Tropical South America		6, 1.25%
South-Eastern Asia		18, 3.75%
South-Central Asia		5, 1.04%
Not specified		98, 20.42%
Length of stay (days)		—
Mean	8.09	
Median	6	
Standard deviation	10.85	
Minimum; maximum	0; 168	
Lethality (*n*, %)	39, 1.95%	16, 3.3%

Total (*n*, %)	2003, 100%	480, 100%

**Table 2:** Prevalence and length of stay considering predefined comorbidities, on univariate and multivariate analysis.

Variables	Prevalence	Mean LOS		Log LOS		Log LOS	
	*N* (%)	Days	*P*	Unadjusted—RR (CI)	*P*	Adjusted—RR (CI)	*P*
Female gender	587 (29%)	8.15 versus 8.07	0.875	0.985 (0.908–1.068)	0.71	0.956 (0.886–1.031)	0.242
Age > 64 (LOS) Age (log LOS)	119 (5.94)	11.2 versus 7.88	0.001	1.005 (1.003–1.007)	<0.001	1.296 (1.116–1.504)	0.001
Malaria related to pregnancy	37 (1.84)	8.41 versus 8.08	0.85	1.049 (0.797–1.381)	0.732		
HIV infection	50 (2.50)	18.4 versus 7.83	<0.001	2.293 (1.813–2.898)	<0.001	1.123 (0.859–1.470)	0.395
Other infections	145 (7.24)	17.93 versus 7.32	<0.001	2.214 (1.927–2.542)	<0.001	1.853 (1.576–2.181)	<0.001
Pneumonia	78 (3.89)	18.3 versus 7.6	<0.001	2.477 (2.054–2.986)	<0.001	1.824 (1.507–2.210)	<0.001
Respiratory failure	89 (4.44)	19 versus 7.6	<0.001	2.284 (1.916–2.724)	<0.001	1.464 (1.212–1.770)	<0.001
Chronic obstructive pulmonary disease	35 (1.75)	12.66 versus 8.01	0.012	1.464 (1.104–1.941)	0.008	1.213 (0.931–1.578)	0.151
Diabetes mellitus	92 (4.60)	9.23 versus 8.04	0.304	1.293 (1.083–1.542)	0.004	1.108 (0.936–1.313)	0.232
Arterial hypertension	139 (6.94)	9.17 versus 8.01	0.226	1.184 (1.024–1.370)	0.023	1.054 (0.918–1.212)	0.457
Renal failure	90 (4.50)	16.1 versus 7.7	<0.001	1.759 (1.474–2.100)	<0.001	1.176 (0.985–1.405)	0.090
Anemia	426 (21.27)	11.8 versus 7.09	<0.001	1.592 (1.458–1.738)	<0.001	1.473 (1.353–1.603)	<0.001
Thrombocytopenia	374 (18.67)	7.37 versus 8.26	0.155	1.078 (0.02–0.170)	0.12		

**Table 3:** Prevalence and lethality considering predefined comorbidities, on univariate and multivariate analysis.

Variables	Prevalence	Lethality				
	*N* (%)	*N* (%)	OR unadjusted (CI)	*P*	OR adjusted (CI)	*P*
Female gender	587 (29%)	9 (1.7%)	0.829 (0.401–1.712)	0.612	0.993 (0.410–2.403)	0.987
Age > 64	119 (5.94)	12 (10)	7.713 (3.802–15.648)	<0.001^†^	5.374 (2.139–13.498)	<0.001
Malaria related to pregnancy	37 (1.84)	2 (5.4)	2.979 (0.691–12.849)	0.161^†^		
HIV infection	50 (2.50)	3 (6)	3.399 (1.011–11.430)	0.071^†^	2.766 (0.477–16.030)	0.256
Other infections	145 (7.24)	10 (6.9)	4.672 (2.230–9.789)	<0.001^†^	2.543 (0.852–7.591)	0.094
Pneumonia	78 (3.89)	9 (11.5)	8.239 (3.767–18.022)	<0.001^†^	1.035 (0.373–2.872)	0.947
Respiratory failure	89 (4.44)	26 (29)	60.349 (29.626–122.935)	<0.001^†^	32.794 (13.163–81.703)	<0.001
Chronic obstructive pulmonary disease	35 (1.75)	0 (0)	—	—	—	—
Diabetes mellitus	92 (4.60)	6 (6.5)	3.970 (1.620–9.730)	0.008^†^	2.103 (0.661–6.694)	0.208
Arterial hypertension	139 (6.94)	5 (3.6)	2.008 (0.773–5.219)	0.188^†^		
Renal failure	90 (4.50)	17 (18.9)	20.017 (10.195–39.302)	0.001^†^	3.590 (1.471–8.759)	0.005
Anemia	426 (21.27)	11 (2.6)	1.466 (0.724–2.970)	0.285		
Thrombocytopenia	374 (18.67)	8 (2.1)	1.127 (0.514–2.471)	0.766		

^†^Fisher exact test due to small expected values.
